# Donor-derived cell-free DNA for detection of acute rejection in lung transplant recipients

**DOI:** 10.3389/fimmu.2025.1531774

**Published:** 2025-01-29

**Authors:** Gökce Yavuz, Julia Walter, Kaimo Hirv, Oliver Wachter, Andrea Dick, Julia Kovacs, Julia Zimmermann, Olaf M. Glueck, Maximilian Vorstandlechner, Nicole Samm, Jan M. Fertmann, Wulf Sienel, Sebastian Michel, Michael Irlbeck, Nikolaus Kneidinger, Rudolf Hatz, Jürgen Behr, Christian Schneider, Teresa Kauke

**Affiliations:** ^1^ Division of Thoracic Surgery, University Hospital, LMU Munich, Munich, Germany; ^2^ Department of Medicine V, University Hospital, LMU Munich, Munich, Germany; ^3^ MVZ Martinsried, Martinsried, Germany; ^4^ Division of Transfusion Medicine, University Hospital, LMU Munich, Munich, Germany; ^5^ Department of Cardiac Surgery, University Hospital, LMU Munich, Munich, Germany; ^6^ Comprehensive Pneumology Center Munich, German Center for Lung Research (DZL), Munich, Germany; ^7^ Department of Anesthesiology, University Hospital, LMU Munich, Munich, Germany; ^8^ Division of Pulmonology, Department of Internal Medicine, Medical University of Graz, Graz, Austria; ^9^ Transplant Center, University Hospital, LMU Munich, Munich, Germany

**Keywords:** ddcfDNA, acute cellular rejection, antibody mediated rejection, allograft injury, lung transplantation, non-invasive biomarker

## Abstract

**Introduction:**

Acute rejection is a significant risk factor for developing chronic lung allograft dysfunction. Current monitoring tools, transbronchial biopsies and HLA antibody determination, have limitations in detecting acute rejection. This study aims to explore the potential utility of donor-derived cell-free DNA (ddcfDNA) as a non-invasive biomarker for detecting acute rejection in lung transplant recipients (LTR).

**Methods:**

We developed a molecular method based on digital droplet PCR to determine the total amount and the proportion of ddcfDNA. Using blood samples collected sequentially post-transplant from a cohort of 81 LTR, we compared median levels of %ddcfDNA in patients with acute cellular rejection (ACR), antibody-mediated rejection (AMR), infection, or decline in pulmonary function (FEV_1_).

**Results:**

Median %ddcfDNA levels were significantly higher in groups with ACR (1.92% [0.70%, 2.30%], p=0.0006), AMR (1.27% [0.34%, 2.29%], p=0.0009), isolated lymphocytic bronchiolitis (0.54% [0.23%, 2.18%], p=0.03), and infection or prolonged ventilation over 30 days (0.50% [0.22%, 2.35%], p=0.005) versus stable allograft function group (0.26% [0.09%, 0.60%]). %ddcfDNA levels were also elevated in patients with FEV1 loss compared to those with stable or improving FEV1 after 12 months (1.98% *vs*. 1.36%, p=0.04). An optimal cut-off of 0.73% for %ddcfDNA was calculated to detect ACR and AMR with 80% specificity and 68% sensitivity.

**Discussion:**

%ddcfDNA is a promising biomarker for identifying allograft injury due to acute rejection in LTR and could be a valuable tool for monitoring allograft health.

## 1 Introduction

Lung transplantation (LTx) has emerged as a life-saving therapeutic option for patients with end-stage lung diseases (1). While significant advancements have been made in surgical techniques, post-transplant care and immunosuppressive therapy, challenges still persist in ensuring favorable long-term success of lung allografts (2, 3). Chronic lung allograft dysfunction (CLAD), as a result of recurrent acute cellular rejection (ACR), antibody-mediated rejection (AMR) or infection, remains a significant hurdle in achieving optimal transplant outcomes (4–10).

The accurate and timely detection of allograft injury, particularly within the first year, is pivotal for implementing targeted therapies to maintain long-term allograft health (11). Diagnostic approaches, such as routine surveillance biopsies and HLA antibody determination, have been integrated in monitoring allograft health (12). The sampling variability of biopsies limits its sensitivity to detect acute rejection and its invasive nature increases the risk of allograft injury (13, 14). HLA antibody determination on the other hand, provides indirect evidence of immune activity and may not correlate with the clinical rejection. These limitations in the early detection of rejection underscore the urgent need for more accurate, quantitative and non-invasive diagnostic methods.

In recent years, a novel biomarker – donor-derived cell-free DNA (ddcfDNA) – has gained attention in the field of solid organ transplantation (SOT) (15, 16). It is released into the recipient’s bloodstream during allograft injury, reflecting the ongoing immunological or inflammatory processes within the transplanted organ. Due to the distinct genetic profiles of donor and recipient, ddcfDNA can be accurately identified in blood plasma using next-generation sequencing, based on single nucleotide and/or insertions-deletions polymorphisms, and quantified by digital droplet PCR (ddPCR) (17–19). Elevated %ddcfDNA levels have been observed in the setting of acute rejection after LTx (20–23) and other SOT (24–27). A routine monitoring of ddcfDNA in kidney transplant recipients enabled early detection of significant graft injury, serving as a valuable prognostic marker and risk assessment tool alongside histology and laboratory surveillance methods post-transplant (28). Several studies have suggested that higher %ddcfDNA levels in the first three months post-LTx can predict poor long-term outcomes and may indicate the potential for chronic lung allograft failure (29, 30). Furthermore, a rise in %ddcfDNA level frequently precedes episodes of AMR, allowing an early detection of acute lung allograft rejection before noticeable changes in lung function occur (31). However, %ddcfDNA has some potential limitations in detecting acute rejection in lung transplant recipients (LTR), as increased %ddcfDNA levels have also been observed in cases of acute respiratory infection and inflammation (32, 33). Furthermore, varying baseline levels between patients may make it challenging to establish a universal threshold for detecting acute rejection. Additionally, %ddcfDNA may not provide information about the type or severity of rejection. Still, its high negative predictive value may be %ddcfDNA`s greatest strength, as it can effectively rule out acute rejection.

In this single-center study, we examined the correlation between %ddcfDNA levels and ACR and AMR within the first year after LTx and assessed the utility of %ddcfDNA analysis in the detection of acute rejection episodes. Furthermore, the study intended to explore the influence of other factors, such as infection, on %ddcfDNA levels. We hypothesize that %ddcfDNA levels will be significantly higher in lung transplant recipients (LTR) experiencing acute rejection and that %ddcfDNA analysis could be a valuable tool completing current diagnostic methods.

## 2 Methods

### 2.1 Study design and population

We prospectively enrolled 81 patients in this study, who underwent double lung transplantation between February 2021 and January 2023 after approval by the Institutional Ethics Committee (project 20-532). This study was conducted in accordance with the Declaration of Helsinki and the informed consent was obtained from each patient for participation.

### 2.2 Sample collection and cfDNA extraction

Plasma samples were collected pre-transplant and post-transplant at day 7, week 2, week 4, week 6, month 3, month 6 and month 12 in two 10ml blood collection tubes (BCT) (Streck). Within a maximum of 48 hours after collection, the BCTs were centrifuged at 1000 g for 20 minutes at 15°C. Subsequently, the plasma supernatant was carefully transferred to a 5mL tube and underwent a second centrifugation at 16000 g for 10 minutes at 15°C. The resulting plasma supernatant was then transferred to a 13ml tube and stored frozen at -80°C until the cfDNA extraction. The extraction of cfDNA was conducted using 4mL of plasma and the QIAamp MinElute ccfDNA Mini Kit (Qiagen), following the manufacturer’s protocol. The elution volume was 30µl. The quantification of cfDNA was performed using the Qubit fluorometer with the Qubit dsDNA HS (High Sensitivity) Assay (Life Technologies). The extracted cfDNA was subsequently stored at -20°C.

### 2.3 Marker determination and quantification with digital droplet PCR

Pre-transplant DNA samples were utilized for determining recipient- and donor-specific genetic markers by using Biotype Multiplex PCR DIPscreen Kit (Biotype). This kit employs Multiplex PCR targeting 33 deletions/insertions of DNA polymorphisms, including the gender-specific marker Amelogenin, facilitating the identification of informative markers, but only 17 markers generate PCR amplicons shorter than 160 bp, making them suitable for cell-free DNA analysis. Subsequently, fragment length analysis was conducted using an ABI3100 Genetic Analyzer (Life Technologies). The ChimerisMonitor 2.0 software from BioType was employed for marker determination to differentiate between the donor and recipient. At least one specific marker was required for patient inclusion in the study. These informative markers were amplified using the MenType DigitalQuant Assay from BioType. The readout was performed using the ddPCR device QX200 Droplet Reader from BioRad. For quantification and data analysis, the QuantaSoft software from BioRad was utilized.

### 2.4 Transplantation management

All patients received standard triple immunosuppression with Tacrolimus (TAC), prednisolone and mycophenolate mofetil (MMF). Pre-immunized patients, those with pre-transplant DSA, received induction therapy with anti-lymphocyte globulin (ALG) for five days beginning on the day of transplantation. Surveillance bronchoscopy with TBBx was performed at 1-, 3-, 6- and 12 months post-transplant. Donorspecific HLA antibody (DSA) was first screened 3-4 weeks post-transplant with quarterly continuation during the first year using Luminex screening and single antigen bead technology. Patients’ sera have been heat-inactivated to avoid prozone effect and were measured undiluted. One or more of the mismatched antigens could explain all donor specificities reported. Specificities with a mean fluorescence intensity of approximately 1.000 were considered positive. Additionally, both procedures were conducted, if patients exhibited clinical signs of rejection or a decline in pulmonary function. All recipients received prophylactic antibacterial, antifungal and antiviral treatment for at least 3 months.

### 2.5 Clinical data collection

The following patient characteristics were documented at baseline for each patient: age, sex, underlying disease, smoking history, cytomegalovirus (CMV) status, HLA of donor and recipient, and whether patients were pre-immunized and received induction therapy. Underlying diseases were categorized as follows: interstitial lung disease (ILD), chronic obstructive pulmonary disease (COPD), autoimmune-related ILD, pulmonary arterial hypertension (PAH) and “others”. The autoimmune-related ILD group included patients with lung diseases resulting from autoimmune-conditions such as sarcoidosis, systemic sclerosis, scleroderma and systemic lupus erythematosus. Patients with cystic fibrosis, bronchiectasis, pulmonary histiocytosis, lymphangioleiomyomatosis and CLAD were categorized in the “others” group. Clinical data documented at time of blood sample collection were laboratory values including CRP, leucocytes and CMV-PCR, microbiologic examination of bronchoalveolar lavage (BAL), candida and aspergillus serology, TBBx histopathology, results of DSA screening and spirometry if present.

### 2.6 Diagnosis of allograft injury

ACR was biopsy-proven and defined according to the International Society of Heart and Lung Transplantation (ISHLT) guidelines. For ACR, perivascular and interstitial mononuclear infiltrates were graded from A1 to A4, with all grades being treated with steroid pulse therapy starting at A1. Lymphocytic bronchiolitis, characterized by bronchiolar inflammation, was diagnosed via TBBx and was graded as B1 or B2 (34). AMR was classified as clinical (definite, probable, possible) or subclinical in accordance with the ISHLT consensus statement (5). Patients received immunoglobulins, if AMR was possible, and additional plasmapheresis and anti-CD20 antibody treatment were applied in cases of probable or definite AMR.

To assess the relationship between %ddcfDNA levels and the observed change in lung function, we categorized patients based on whether they experienced a decline in forced expiratory volume in 1 second (%FEV_1_) or not. We quantified the change in %FEV_1_ by calculating the difference between the initial spirometry conducted within the first 3 months after LTx and the spirometric assessments carried out at 6-month and 12-month intervals thereafter.

After analyzing factors affecting %ddcfDNA levels, we determined that respiratory infection, invasive ventilation and prolonged stay in the Intensive Care Unit (ICU) > 30 days post-transplant were factors causing %ddcfDNA elevation. Therefore, we summarized these factors into a category of competing-risk events for further analysis. Respiratory infection was defined by a combination of clinical symptoms including cough, fever, dyspnea, elevated inflammatory values (CRP and leukocytes), positive imaging findings (lung infiltrates, pleural effusion), and positive microbiological assessment from bronchoalveolar lavage or serology, and was treated with anti-infective drugs.

### 2.7 Categorization of samples

%ddcfDNA measurements of plasma samples collected within 14 days post-transplant were excluded from the final analysis to eliminate the influence of ischemia-reperfusion and surgical trauma. Each %ddcfDNA measurement was categorized based on the results of diagnostic tools and clinical information into the following groups; ACR, AMR, isolated lymphocytic bronchiolitis (LB, B1/B2 in TBBx), competing-risk events, and stable allograft function. Only %ddcfDNA samples collected a maximum of 3 days before the follow-up assessment were included in the analysis.

### 2.8 Statistical analysis

The aim of our analysis was to determine a possible impact of the independent variable ddcfDNA on the clinical outcomes after lung transplantation. Therefore, we conducted the following statistical analyses. We presented patient characteristics as absolute and relative frequencies for categorical variables. To discern discrepancies in the frequency distributions between patients experiencing either ACR or AMR and patients with stable allograft function, we employed Chi-squared test or, in cases where cell numbers were less than six, the Fisher exact-test. For numerical variables with normal distribution, we provided the means along with their corresponding standard deviations (sd). Conversely, for numerical variables that did not conform to a normal distribution, we presented the median values alongside the first quartile (Q1) and third quartile (Q3) values. To determine the normality of numeric variables, we conducted visual assessments using QQ-plots and histograms as well as Shapiro-Wilks test. In instances of two-group comparisons for normally distributed variables, we employed Student’s t-tests. Conversely, for variables that did not meet the normality assumption, we resorted to the Wilcoxon-Rank sum test. When our analyses extended to more than two groups, we employed ANOVA for normally distributed variables and the Kruskall-Wallis test for those that deviated from normal distribution. To investigate the relationship between the %ddcfDNA and CRP or leukocyte levels, we utilized the Pearson correlation coefficient. We used receiver operating characteristic curves (ROC) to determine optimal cut-offs of %ddcfDNA to identify patients with AMR or ACR and patients with stable allograft function. Optimal cut-offs are presented alongside area under the curve (AUC), sensitivity, specificity, negative predictive value (NPV), and positive predictive value (PPV). We determined statistical significance in all analysis using two-sided p-values with alpha errors <0.05. We used R Version 4.0.0 and RStudio Version 1.4 to perform the data analysis and created tables and figures in RStudio and Microsoft Excel. Cut-offs from ROC were determined using IBM SPSS Statistics for Windows, Version 29.0.

## 3 Results

### 3.1 Study population

107 patients underwent lung transplantation in our clinic between February 2021 and January 2023 and were screened for inclusion in this study. 26 patients had to be excluded because of incomplete follow-up (n=13), early death (n=5), no available informative marker (n=6) and multi-organ or single lung transplantation (n=2), In total 81 patients have been included in the final analysis. Of these, 14 patients (17.3%) showed an episode of ACR, 12 showed (14,8%) an episode of AMR and 2 experienced (2,5%) both ACR and AMR during the follow-up period of 12 months. 3 of the ACR episodes were grade A2 and 13 of them were grade A1. 70.4% of patients were male, the mean age was 55.4 years (sd = 11.0). The most common underlying disease was ILD (58%) followed by COPD (16%) and autoimmune-related ILD (9.9%). There was no significant difference in age (p=0.38) and gender distribution (p=0.61) between patients with or without rejection. There was a significant difference in pre-immunization status, with more patients who had an acute rejection and being pre-immunized (p = 0.02, **Table 1**).

**Table 1:** Patients characteristics stratified by development of acute rejection.

	all patients (n = 81)		acute rejection (ACR and AMR) (n = 28)		no rejection (n = 53)		p-value
	mean	sd	mean	sd	mean	sd	
age in years	55.4	11.0	56.8	10.1	54.6	11.5	0.38
BMI	23.0	3.2	23.4	2.9	22.8	3.4	0.41
	n	%	n	%	n	%	p-value
sex							
male	57	70.4%	21	75.0%	36	67.9%	
female	24	29.6%	7	25.0%	17	32.1%	0.61
underlying disease							
ILD	47	58.0%	17	60.7%	30	56.6%	
COPD	13	16.0%	3	10.7%	10	18.9%	
auto-immune related ILD	8	9.9%	4	14.3%	4	7.5%	
PAH	3	3.7%	1	3.6%	2	3.8%	
other	10	12.3%	3	10.7%	7	13.2%	0.78
smoking status							
former	45	55.6%	17	60.7%	28	52.8%	
never	36	44.4%	11	39.3%	25	47.2%	0.66
CMV status							
D-R-	22	27.2%	7	25.0%	15	28.3%	
D-R+	14	17.3%	3	10.7%	11	20.8%	
D+R-	21	25.9%	8	28.6%	13	24.5%	
D+R+	24	29.6%	10	35.7%	14	26.4%	0.64
pre immunization							
yes	8	9.9%	6	21.4%	2	3.8%	
no	73	90.1%	22	78.6%	51	96.2%	**0.02**

Patient characteristics as means with standard deviation for numerical, absolute and relative frequencies for categorical variables. Comparison of mean values with Student’s t-test, and comparison of frequencies with Chi^2^-test or fishers exact test depending on sample size.

BMI, body mass index; CMV, cytomegalovirus; COPD, chronic obstructive pulmonary disease; D, donor; ILD, interstitial lung disease; PAH, pulmonary arterial hypertension; R, recipient; SD, standard deviation.

Bold values are indicating statistically significant variables.

### 3.2 Association of %ddcfDNA and identified competing-risk events

We found significant differences between median values of %ddcfDNA in patients with and without respiratory infection (1.40% [0.40%, 2.70%] *vs*. 0.99% [0.30%, 2.40%], p = 0.02), patients with and without ventilation time > 30 days (1.46% [0.78%, 3.70%] *vs*. 1.00% [0.30%, 2.30%], p = 0.001), and patients with and without prolonged ICU stays > 30 days (1.46% [0.60%, 3.30%] *vs*. 0.90% [0.30%, 2.30%], p = 0.001). No significant differences were observed regarding ECMO, surgical revision, CMV reactivation and pre-immunization (**Table 2**). As a result, we categorized samples collected from patients with respiratory infection, prolonged ventilation time and ICU stay > 30 days, into a group competing-risk events for further analysis.

**Table 2:** Comparison of median values of %ddcfDNA across confounding factors.

	mean	sd	median	IQR	p-value
respiratory infection	2.45	3.43	1.40	[0.4, 2.7]	**0.02**
no respiratory infection	2.17	4.17	0.99	[0.3, 2.4]	
ECMO	2.27	4.27	1.00	[0.4, 2.6]	0.93
no ECMO	2.27	3.50	1.21	[0.3, 2.5]	
ventilation time > 30 days	3.44	5.68	1.46	[0.78, 3.7]	**0.001**
ventilation time <= 30 days	1.95	3.30	1.00	[0.3, 2.3]	
ICU stay > 30 days	3.16	5.20	1.46	[0.6, 3.3]	**0.001**
ICU stay <= 30 days	1.92	3.29	0.90	[0.3, 2.3]	
surgical revision	2.43	4.36	1.30	[0.4,2.8]	
no surgical revision	2.20	3.74	1.00	[0.31,2.40]	0.22
CMV reactivation/infection	2.73	4.80	1.26	[0.32,2.73]	0.09
no CMV reactivation/infection	1.63	2.07	0.90	[0.32,2.27]	
CMV recipient positive	2.19	3.02	1.20	[0.4, 2.6]	0.11
CMV recipient negative	2.35	4.73	1.00	[0.29, 2.3]	
pre-immunized	2.97	4.70	1.25	[0.5, 2.4]	0.24
non-immunized	2.19	3.84	1.10	[0.3, 2.5]	

Comparison of mean and median values with standard deviation and Q1 and Q3 of %ddcfDNA across different confounding factors. P-values from Wilcoxon rank sum test. CMV, cytomegalovirus; ECMO, extracorporeal membrane oxygenation; ICU, intensive care unit.

Bold values are indicating statistically significant variables.

### 3.3 Correlation of %ddcfDNA and lung allograft function groups

When we categorized the samples according to results of TBBx and DSA screening, we observed notable disparities. Specifically, %ddcfDNA levels were significantly higher in the group with A1 or A2 than the group with A0 in TBBx (1.85% [0.78%, 2.00%] *vs*. 0.40% [0.15%, 1.60%], p = 0.04). Additionally, %ddcfDNA levels were significantly elevated in the group with *de novo* donor-specific HLA antibodies (dnDSA) compared to the group without (1.32% [0.35%, 2.40%] *vs*. 0.32% [0.15%, 0.91%], p = 0.01) (**Table 3**).

**Table 3:** Comparison of median values of %ddcfDNA regarding lung allograft function and spirometry results.

Categorization of samples	mean	sd	median	IQR	p-value
ACR	1.83	1.19	1.92	[0.70,2.30]	
AMR	2.30	3.19	1.27	[0.34,2.29]	
isolated LB (B1,B2)	1.41	1.94	0.54	[0.23,2.18]	
competing-risk events	1.80	3.53	0.50	[0.22,2.35]	
stable allograft function	0.69	1.12	0.26	[0.09,0.60]	**0.0003**
dnDSA	2.50	3.36	1.32	[0.35,2.40]	
no dnDSA	1.13	2.47	0.32	[0.15,0.91]	**0.01**
A1-A2 (TBBx)	1.47	0.76	1.85	[0.78,2.00]	
A0 (TBBx)	1.37	2.66	0.40	[0.15,1.60]	**0.04**
Categorization of patients					
%FEV_1_ 12 months					
stable or improvement	1.36	2.75	0.64	[0.27,1.66]	
decline	1.98	2.5	1.70	[0.50,2.30]	**0.04**
%FEV_1_ 6 months					
stable or improvement	1.97	3.59	1.1	[0.34,2.38]	
decline	2.09	2.52	1.36	[0.40,2.30]	0.38

Comparison of mean and median values with standard deviation and Q1 and Q3 of %ddcfDNA across different lung allograft function groups and regarding to change in spirometry results. P-values from Wilkoxon rank sum test and Kruskall-Wallis test. ACR, acute cellular rejection; AMR, antibody mediated rejection; dnDSA, *de novo* donor-specific HLA antibody; FEV_1_, forced expiratory volume in 1 second; TBBx, transbronchial biopsy.

Bold values are indicating statistically significant variables.

**Figure 1:**
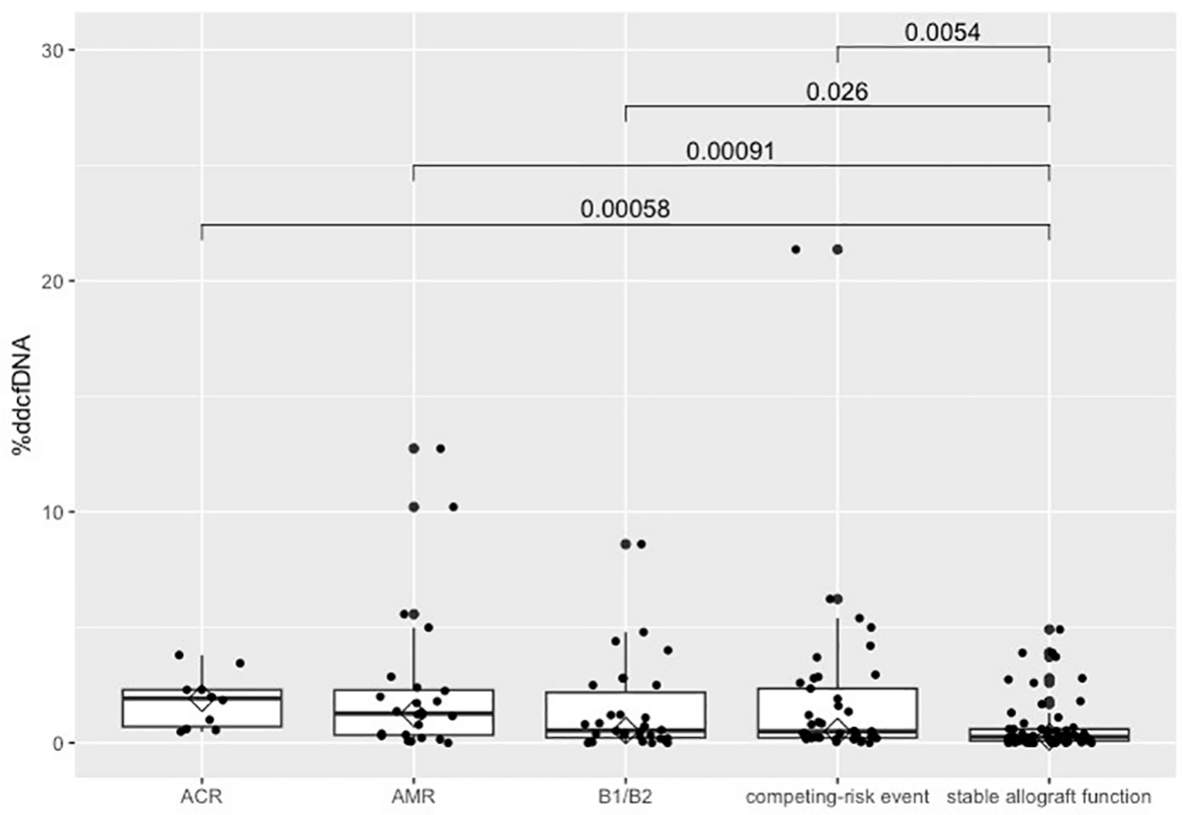
Comparison of %ddcfDNA according to graft function. Boxplot of %ddcfDNA levels by categories of lung allograft function. P-value from Kruskall-Wallis test. %ddcfDNA, percentage of donor-derived cell-free DNA; B1/B2, lymphocytic bronchiolitis.

**Figure 2:**
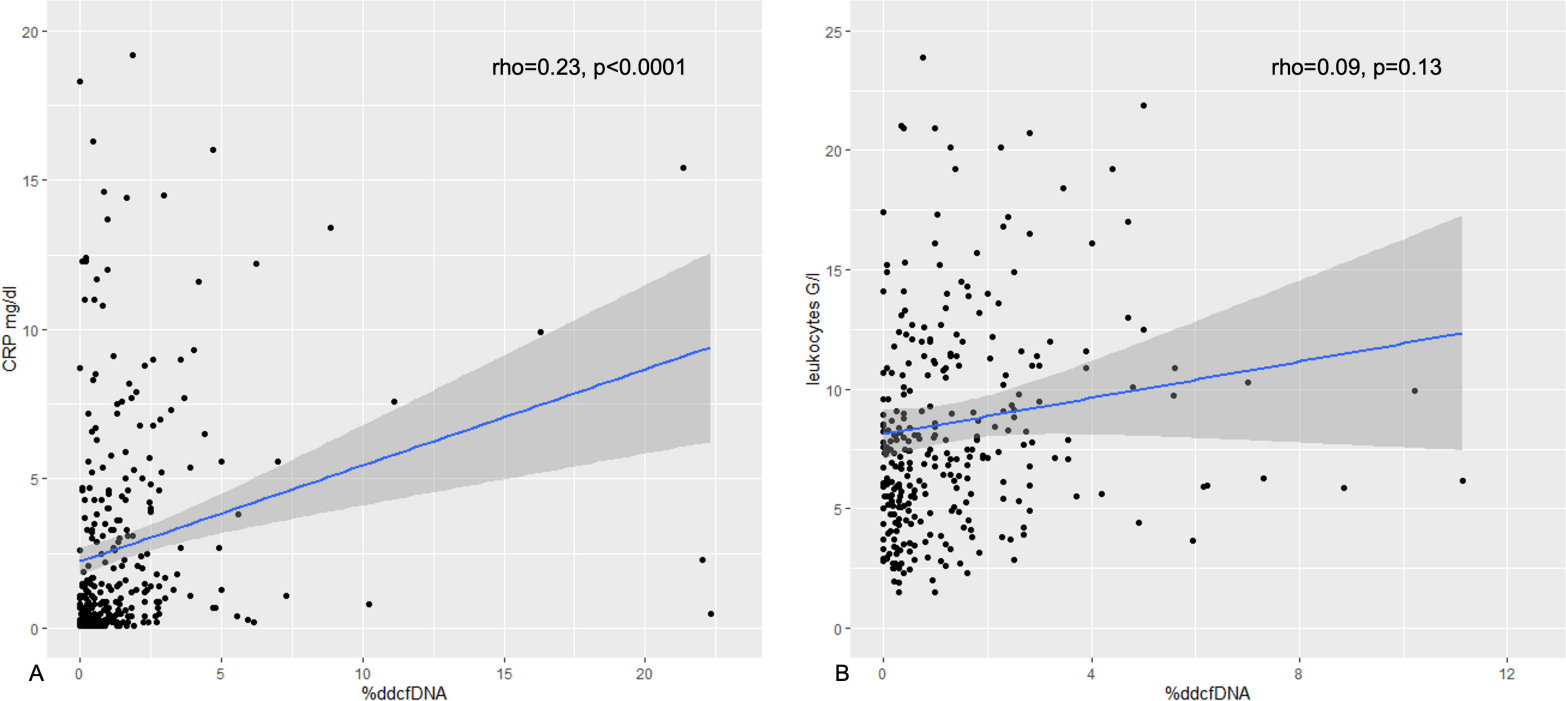
Correlation of %ddcfDNA with CRP and leukocytes. Scatterplot of %ddcfDNA and **(A)** CRP, and **(B)** leukocytes with Pearson correlation coefficient and p-value. %ddcfDNA, percentage of donor-derived cell-free DNA; CRP, C-reactive protein.

When categorizing samples into five groups we found an overall highly significant difference (p=0.0003) in median %ddcfDNA. Specifically, median %ddcfDNA levels were significantly elevated in the group with ACR (1.92% [0.70%, 2.30%], p = 0.0006), AMR (1.27% [0.34%, 2.29%], p = 0.0009), isolated LB (0.54% [0.23%, 2.18%], p = 0.03), and competing-risk events (0.50% [0.22%, 2.35%], p = 0.005) compared to those with stable allograft function (0.26% [0.09%, 0.60%]) (**Figure 1**, **Table 3**). %ddcfDNA levels remained significantly higher in AMR group, even when the %ddcfDNA measurement was conducted 7 days before DSA determination (p=0.002). Matching the inflammatory reaction during respiratory infection or acute rejection, a significant moderate correlation was observed between %ddcfDNA and CRP levels (r=0.23, p<0.0001) over all measurements (**Figure 2**). Leukocyte levels were not significantly correlated to %ddcfDNA.

When we categorized the patients according to the %FEV_1_ trend in spirometry, we have observed that median %ddcfDNA levels were significantly higher in patients with %FEV_1_ decline compared to those with stable or improving lung function after 12 months (1.98% *vs*. 1.36%, p=0.04). Median levels of %ddcfDNA were not significantly different after 6 months in patients with %FEV_1_ decline and stable or improving lung function (1.13% *vs*. 1.10%, p = 0.38, **Table 3**).

### 3.4 Optimal cut-off for %ddcfDNA

**Figure 3:**
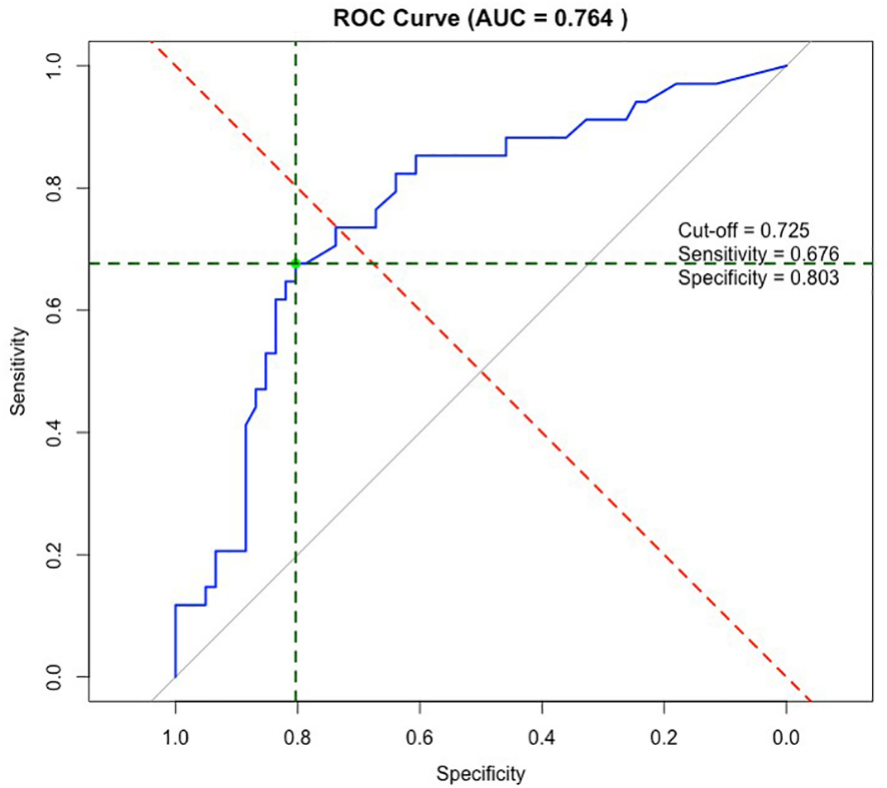
ROC-curve of %ddcfDNA and acute rejection. ROC-curve of %ddcfDNA and acute rejection (ACR and AMR) with AUC of 0.76. Optimal cut-off for %ddcfDNA of 0.73% with 68% sensitivity, 80% specificity, PPV of 66% and NPV of 82%. ROC, receiver operating characteristics; AUC, area under the curve; PPV, positive predictive value; NPV, negative predictive value.

Using ROC-curve we established that the optimal cut-off of %ddcfDNA to distinguish between patients with stable allograft function and patients with acute rejection (ACR and AMR) was 0.73%. The AUC for this cut-off was 0.76 with a sensitivity of 68% and a specificity of 80%, a positive predictive value of 66%, and a negative predictive value of 82% (**Figure 3**).

## 4 Discussion

We analyzed %ddcfDNA levels in recipient’s blood post-transplant after double LTx at regular intervals during the first year to identify the correlation between %ddcfDNA and allograft injury due to rejection or/and infection. For the measurement of %ddcfDNA levels, we used Biotype Multiplex PCR DIPScreen Kit and MenType DigitalQuant Assay, as these kits were already in the laboratory for chimerism monitoring following hematopoietic stem cell transplantation. Typically, two informative markers were used for each patient. The Biotype Kit targets 33 polymorphisms, but only 17 markers generate PCR amplicons shorter than 160 bp, making them suitable for cell-free DNA analysis. Due to the limited number of short targets, it was not always possible to identify two informative markers for every patient. For 16 patients only one informative marker was available, while no informative markers were available for six patients. To avoid excluding patients due to the lack of informative markers, future studies should prioritize methods that include a higher number of markers.

Our study was the first to conduct an analysis to identify factors affecting ddcfDNA levels other than rejection or infection such as ECMO or ventilation time as proposed by Pedini (35). We detected significantly higher %ddcfDNA levels in patients with respiratory infection, prolonged ventilation time, and ICU stay exceeding 30 days, categorizing them into a group with competing-risk events. Reperfusion edema or respiratory infection, necessitating high-pressure ventilation can result in extended ICU stay. Prolonged ventilation time and recurrent respiratory infections can cause cell damage and apoptosis, reflected by elevated %ddcfDNA.

In subsequent analysis involving five sample categories, we have detected significantly higher (almost 5- and 7-fold higher, respectively) %ddcfDNA in the group with AMR and ACR episodes, compared to those with stable allograft function. This was slightly higher than in recent study findings reporting 3- to 5-fold higher levels (22, 23, 31). Through our initial analysis, we were able to eliminate the samples with elevated %ddcfDNA levels collected during competing-risk events such as respiratory infection and high-pressure ventilation. Therefore, the median %ddcfDNA level of our stable cohort was lower compared to other study findings, resulting in a difference in fold. We exclusively included %ddcfDNA measurements within a maximum timeframe of 3 days from the follow-up assessment due to short half-life of cfDNA. Nevertheless, the group with AMR exhibited significantly higher %ddcfDNA levels even within a 7-day timeframe, highlighting the capability of ddcfDNA analysis for earlier detection of AMR before determination of DSA likewise suggested by Jang and Agbor-Enoh (17, 29, 31).

Notably, elevated %ddcfDNA levels were observed in the group with isolated LB in TBBx or with competing-risk events, underlining the fact that ddcfDNA is a marker of graft damage independent from its cause. Our study is the first one presenting significantly higher %ddcfDNA levels in patients with isolated LB as expected by Khush (20), since LB is likely to be an independent risk factor for developing CLAD (36, 37). A significant moderate correlation between %ddcfDNA and CRP was noted similar to other studies (19). Consistent with these findings, patients with FEV1 decline after 12 months showed significantly higher %ddcfDNA compared to patients with stable or improving pulmonary function.

An optimal cut-off of 0.73% for %ddcfDNA was calculated to detect acute rejection (ACR and AMR) with a specificity of 80%, a sensitivity of 68%, a positive predictive value (PPV) of 66%, and a negative predictive value (NPV) of 82%. However, these results still indicate room for improvement. Other studies have reported cut-off values of 1% (23, 33) or 0.85% (20, 22) with comparable levels of specificity and sensitivity. The reported sensitivity of 68% implies that 32% of patients with rejection were false negatives and missed based on our calculated cut-off value. This limitation has important clinical implications, as undiagnosed cases of rejection may progress undetected, potentially affecting patient outcomes. Furthermore, the relatively low PPV of 66% indicates that a significant proportion of patients testing positive for rejection may not actually have the condition. Both low PPV and sensitivity could lead to missed cases of rejection. To avoid this, a two-threshold system could be applied as suggested by Halloran et al. (38). An increase in ddcfDNA levels due to rejection could be masked by elevated recipient cfDNA levels resulting from infection or inflammation in other organ systems, potentially lowering the ddcfDNA ratio (%ddcfDNA) below the threshold. Therefore, calculating a cut-off value for absolute ddcfDNA levels and combining it with %ddcfDNA threshold, may improve sensitivity for detecting acute rejection. Nevertheless, due to its high NPV, %ddcfDNA levels below the cut-off indicate stable allograft function, potentially reducing the need for surveillance biopsies. However, levels above cut-off, given the specificity of 80%, necessitate additional diagnostics, such as biopsies and HLA antibody determination, to identify the cause of allograft injury. Challenges in mapping the complexity of immune response highlight the importance of developing an algorithm by combining ddcfDNA with other clinical parameters and current diagnostic tools to provide greater robustness in prediction of rejection. Routine screening for DSA, protocol biopsies and T-cell monitoring have been part of our diagnostic options for years. The measurement of ddcfDNA might be an additional tool for risk assessment as it provides very early information about the organ-specific damage in order to support clinicians in identifying patients at higher risk for rejection.

Limitations of our study include single-center experience and incomplete data regarding %ddcfDNA course during or after treatment for rejection episodes, since we collected samples at specific intervals rather than directly post-treatment. Additionally, some measurements had to be excluded from statistical analysis due to long interval between %ddcfDNA sample collections and follow-up assessments, leading to a limited sample size in some cohorts.

The utilization of %ddcfDNA offers a non-invasive method to assess allograft health. Monitoring %ddcfDNA levels starting 2 weeks post-transplant, with subsequent measurements every 4 weeks during the first 3 months, every 6 weeks from 3 to 6 months, and every 3 months thereafter for up to 2 years, along with intensified monitoring in cases of respiratory complications could provide valuable insights into allograft health. With a turnaround time of 3 days for regular and 24 hours for urgent analyses, the implementation of ddcfDNA analysis into clinical practice may enable timely interventions. Moreover, %ddcfDNA analysis allows accurate detection of rejection episodes when used in combination with current diagnostic tools.

Despite its potential, challenges exist, such as standardized protocols and cut-off values for interpreting the results, as an elevation of %ddcfDNA can also occur due to infection and inflammation. Accurate interpretation of %ddcfDNA analysis results requires additional clinical information, including laboratory values, radiological imaging, and spirometry results. Further multi-center, observational studies are essential for validation of %ddcfDNA as a reliable marker for detecting rejection and to determine the approach for integrating this biomarker into routine monitoring. Additionally, future studies should investigate the utility of %ddcfDNA in guiding adjustments to immunosuppressive treatment and evaluating therapeutic outcomes in patients diagnosed with ACR and AMR. In conclusion, the integration of %ddcfDNA analysis into the diagnostic tools for monitoring allograft health alongside histology and DSA determination exhibits considerable promise.

## Data Availability

The raw data supporting the conclusions of this article will be made available by the authors, without undue reservation.
