# Supplementary material for: Donor-derived cell-free DNA for detection of acute rejection in lung transplant recipients
Source: Front Immunol. 2025 Jan 29;16:1531774. doi: 10.3389/fimmu.2025.1531774 (PMC11814210; doi:10.3389/fimmu.2025.1531774)
Supplement: Supplementary file 1 [file DataSheet1.pdf]

Patient ID	First detection post Tx in days	HLA Class (I=1, II=2, both=3)	DSA	Minimum MFI
2	20	2	DQ2	1200
3	14	1	Cw10	2200
4	87	2	DQA1	16000
6	37	2	DQA1	5000
14	13	1	B8	3000
16	14	2	DQ7,DQ8,DQ9	6000
22	203	2	DQ6,DQ7	23000
76	17	3	A1, A2, DPA1*02, DQA1*05:01	5000
81	9	2	DQ7	2500
84	10	1	B49, Bw4	14000
88	20	3	B27, Cw4, DR13	9000
99	27	2	DQ8	2000
104	34	2	DQ6, DQA1*01	6000
108	18	1	Cw7	9000

Table: Patients with DSA post-transplantation including information about the time of first DSA detection post-transplant, HLA class and minimum MFI.
